# HEALTH-SEEKING BEHAVIOR OF COMMUNITY-LIVING OLDER ADULTS RELATES TO AWARENESS OF COMMUNITY SERVICES

**DOI:** 10.1093/geroni/igad104.2897

**Published:** 2023-12-21

**Authors:** Denise Connelly, Navjot Gill, Marla Beauchamp, Laura Brunton

**Affiliations:** Western University, London, Ontario, Canada; University of Waterloo, Waterloo, Ontario, Canada; McMaster University, Hamilton, Ontario, Canada; Western University, London, Ontario, Canada

## Abstract

Research has shown that older adults desire to age in their homes. To age at home safely and comfortably, there are a variety and number of community support services available, however, previous studies show older adults do not utilize these services fully. There is a need for clarity about this gap between availability and use. Understanding the construct of health-seeking behaviour by community-living older adults may provide insights into this identified gap. Therefore, the objective was to determine whether health-seeking behaviour in community-living older adults is related to: dimensions of wellness; awareness of community support services; living as an older man or woman; or being younger or older than 75 years. A cross-sectional study design involving 99 community-living older adults (≥ 65 years, without impaired executive function) employed validated outcome measures of physical function, fear of falling, personal resilience, social well-being, spiritual well-being, awareness of community support services, and health-seeking behaviour. Relationships were calculated using multiple linear regression. Health-seeking behaviour was positively correlated (R= 0.40, p<.01) with awareness of community support services. Health-seeking behaviour was not correlated with dimensions of wellness (i.e., physical function, fall risk, personal resilience, spiritual wellbeing, or social wellbeing). No difference between age groups (U=986.5, n1=68, n2=31, p=0.61) nor sex (U=931.5, n1=66, n2=33, p=0.24) was found for health-seeking behaviour. The findings indicate that awareness of community support services positively influenced health-seeking behaviour. The healthcare system should ensure equitable, accessible, age-friendly, and relevant information and distribution strategies to increase awareness of older adults for local supportive services.

